# Magnetic resonance spectroscopic evidence of increased choline in the dorsolateral prefrontal and visual cortices in recent onset schizophrenia

**DOI:** 10.1016/j.neulet.2021.136410

**Published:** 2021-12-18

**Authors:** Jason Smucny, Cameron S. Carter, Richard J. Maddock

**Affiliations:** Department of Psychiatry and Behavioral Sciences, University of California, Davis, 2230 Stockton Blvd., Sacramento, CA 95617, USA

**Keywords:** Cortex, Psychosis, Glutamate, MRS, NAA, Occipital

## Abstract

A complete characterization of neurometabolite profiles in the dorsolateral prefrontal cortex (DLPFC) in recent onset schizophrenia (SZ) remains elusive. Filling in this knowledge gap is essential in order to better understand how the neurochemistry of this region contributes to SZ pathology. To that end, DLPFC N-acetyl aspartate (NAA), myo-inositol, glutamate, choline, and creatine levels were examined by 3 T magnetic resonance spectroscopy (MRS) in recent onset individuals with SZ (*n* = 40) and healthy controls (HC) (*n* = 47). Metabolite levels were also examined in the visual cortex (VC) as a control region. People with SZ showed significantly higher choline in both the DLPFC and VC, but no differences in NAA, myo-inositol, glutamate, or creatine in either region. A trend-level negative correlation was also observed between DLPFC NAA and negative symptoms in SZ. Our results suggest that choline is increased in both the prefrontal and occipital cortices in recent onset SZ, and that DLPFC NAA levels may be inversely related to negative symptoms in the illness. The observed increase in choline-containing compounds in both DLPFC and VC in recent onset SZ could reflect increased membrane remodeling such as occurs in activated microglia and astrocytes in response to neuroinflammation.

## 1. Introduction

Multiple lines of evidence suggest that the dorsolateral prefrontal cortex (DLPFC) is abnormal in schizophrenia (SZ). Behaviorally, it is well established that cognitive processes associated with the region are impaired in SZ, such as cognitive control and working memory [22,31]. Neuroimaging studies further suggest these regions are functionally abnormal in SZ during tasks that measure activation during these cognitive processes [25]. Postmortem studies demonstrating morphological abnormalities in pyramidal cells and inhibitory interneurons further suggest that the DLPFC is altered on a microscopic level, and that these changes contribute to its functional pathology [12,31].

Given the nature of these abnormalities, one may speculate that the DLPFC is also altered on a neurochemical level in SZ. Indeed, as first demonstrated in a study by Bertolino [8] and subsequently shown by *meta*-analysis [36], evidence suggests that the illness is associated with reduced levels of N-acetyl aspartate (NAA), a putative marker of neuronal integrity and metabolism [26]. Meta-analyses of DLPFC glutamate, in contrast, have reported no difference in levels of the excitatory neurotransmitter between patients and controls [17,30]. As demonstrated in these *meta*-analyses, however [17,30,36], the great majority of DLPFC MRS studies in SZ to date have been conducted in chronic patients. The biochemical make-up of the DLPFC in recent onset and/or first episode SZ is therefore comparatively less well studied.

Here we report on levels of five reliably measured brain metabolites in the DLPFC in individuals with recent onset SZ and healthy control subjects. Specifically, we measured the content of NAA, glutamate (essential roles in both neurotransmission and cellular metabolism), myo-inositol (neuronal and glial osmolyte that is produced by recycling of inositol phosphate second messengers and de-novo synthesis from glucose; elevated in gliosis [14,37]), choline-containing compounds (putative index of cell membrane metabolism [28]) and creatine (carrier molecule for high energy phosphate bonds in all brain cell types [23]). As a control region, we also examined levels of these metabolites in the visual cortex (VC).

## 2. Methods

### 2.1. Participants

50 HCs and 41 individuals with recent-onset SZ-spectrum disorders (including SZ, schizoaffective disorder, and schizophreniform disorder, who were combined into a single “SZ” group) were recruited from the UC Davis Early Psychosis Programs (EDAPT and SacEDAPT Clinics) for a study examining neuroinflammatory processes in SZ [21]. Of these, DLPFC voxel acquisitions were available for 46 HCs and 40 SZ individuals, and VC voxel acquisitions for 44 HCs and 40 people with SZ. All individuals were between 14 and 30 years of age. SZ-spectrum participants were scanned within two years of their first psychotic episode (including duration of untreated psychosis). The University of California, Davis Institutional Review Board approved the study. Participants gave written informed consent and were paid for their participation.

Patients were assessed with the Structured Clinical Interview for DSM-IV-TR (SCID) [13]. Patients were excluded for a diagnosis of major medical or neurological illness, head trauma, substance abuse in the previous 3 months (or a positive urinalysis on the day of scanning), Weschler Abbreviated Scale of Intelligence-2 score (WASI) [35] score < 70, and magnetic resonance imaging (MRI) exclusion criteria (e.g. claustrophobia, metal in the body). Control subjects were excluded for all of the above as well as a history of Axis I mental illness or first-degree family history of psychosis.

### 2.2. Clinical assessments

Consistent with prior work [6], three core symptom dimensions were calculated. “Poverty” combined emotional withdrawal, motor retardation, and blunted affect from the Brief Psychiatric Rating Scale (BPRS) [34] with anhedonia/asociality, avolition/apathy, alogia, and affective flattening from the Scale for the Assessment of Negative Symptoms (SANS) [4]. “Disorganization” combined conceptual disorganization, mannerisms and posturing, and disorientation scores from the BPRS with attention score from the SANS as well as positive formal thought disorder, and bizarre behavior scores from the Scale for the Assessment of Positive Symptoms (SAPS) [5]. “Reality distortion” combined grandiosity, suspiciousness, hallucinations, and unusual thought content from the BPRS with hallucinations and delusions from the SAPS [6]. Functioning was assessed using the Global Assessment of Function (GAF) [2]. Chlorpromazine equivalent antipsychotic doses were calculated using published guidelines for conventional [3] and atypical [38] antipsychotics.

### 2.3. Scanning parameters

**Fig. 1.**
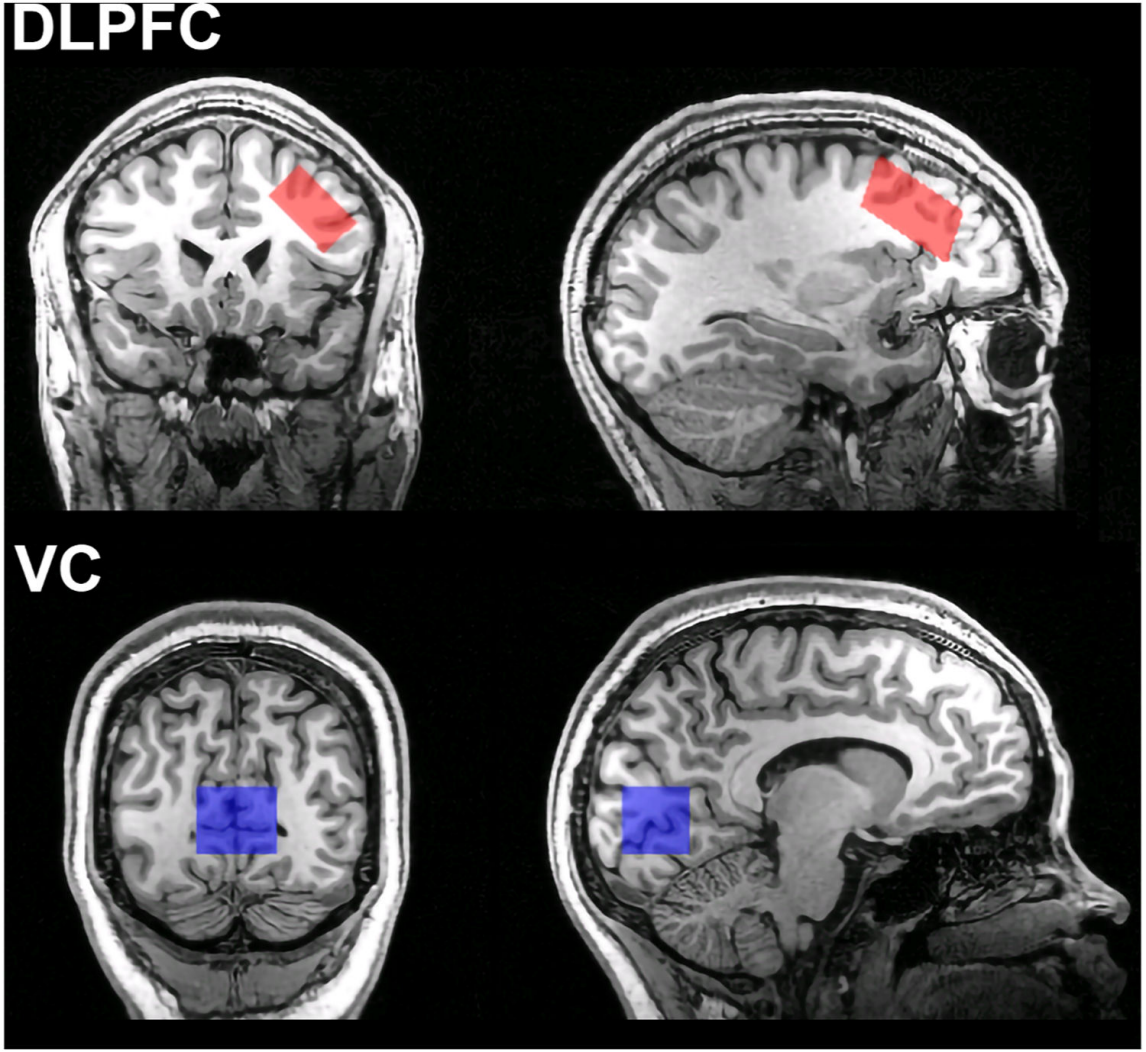
Representative voxel locations for the dorsolateral prefrontal cortex (DLPFC) (top) and visual cortex (VC) (bottom).

Imaging data were obtained using a 3 T Siemens Tim Trio MRI scanner with a 32 channel head coil. T1-weighted MPRAGE structural images were acquired with the following settings: TR = 2530-msec, echo time = 3.5-msec, flip-angle = 7°, field of view = 256 mm, 1 mm isotropic voxels. Water suppressed 1H-MRS data were acquired from voxels placed in the left DLPFC and VC using a PRESS sequence with the following parameters: TE/TR = 30/1500; bandwidth = 2000 Hz; delta frequency = −1.7 ppm, NEX = 160 scans, duration = 240 s. Water non-suppressed spectra were acquired from the same location with the same scanning parameters except NEX = 16. The DLPFC voxel was 15.75 cc in volume (30 × 15 × 35 mm) and was placed in the left middle frontal gyrus over Brodmann areas 9 and 46, angled to be parallel to the brain surface (Fig. 1). The visual cortex voxel was 18.75 cc in volume (30 × 25 × 25 mm) and was centered on the midline over the calcarine fissures bilaterally with its posterior face 8 mm anterior to the posterior limit of the occipital cortex (Fig. 1).

### 2.4. MRS data analysis and quality control

MRS spectra were analyzed using LCModel 6.3-1L [29]. Water-suppressed individual spectra were fit within an analysis window of 4.0 to 1.7 ppm and a basis set matched to the scanning parameters provided with LCModel (consisting of the following: creatine, phosphocreatine, NAA, n-acetylaspartylglutamate (NAAG), phosphocholine, glycerophosphorylcholine, myo-inositol, glutamate, glutamine, glutathione, scyllo-inositol, aspartate, taurine, GABA, and glucose. The PRESS voxels were segmented using the MPRAGE images, the FAST algorithm in FSL [39] and in-house software to estimate the CSF, gray matter and white matter fractions. Fitted NAA, glutamate, myo-inositol, total choline (phosphocholine + glycerophosphorylcholine), and total creatine (creatine + phosphocreatine) levels for each voxel were normalized to the unsuppressed water signal, incorporating corrections for the fractional volumes of CSF, gray matter, and white matter in the voxel (as described in equation 3 of the recent consensus paper by Near, et al. [27]). Analyses were also performed in which the same metabolites other than creatine were normalized to total creatine.

A recent *meta*-analysis showed that use of strict quality thresholds for inclusion of MRS data increases sensitivity for demonstrating metabolite abnormalities in studies of schizophrenia [25]. Thus, rigorous quality control procedures were performed prior to between group analyses. First, spectra were visually inspected for distortion of baseline or peaks suggesting significant movement during scanning. Next, spectral quality was assessed by LCModel-calculated spectral line width (FWHM) and signal-to-noise ratio (SNR). For line width, individual spectra with FWHM > 0.06 ppm and/or FWHM > 3 SD above the overall mean across all participants were excluded. For SNR, spectra with SNR < 20 or > 3 SD below the overall mean were excluded. Spectral data for a voxel were also excluded if the tissue fraction of gray matter (T%GM calculated as %GM/(%GM+%WM)) was not within 3 SD of the mean for that voxel across all participants. Finally, individual metabolite data were excluded if Cramer-Rao lower bound values were ≥ 15 or if a metabolite measurement was not within 3 SD of the within-group mean for that voxel. A representative spectrum is shown in the Supplement.

To compare metabolite levels for the DLPFC and VC voxels between groups, a univariate ANCOVA analysis was performed in SPSS v. 27 (IBM) with diagnosis (HC vs. SZ) as a fixed factor, T%GM as a covariate, and metabolite concentration as the dependent variable. T%GM fraction was included as a covariate because metabolite values may correlate with the relative amount of gray matter in the tissue fraction of the voxel [32]. *P* < .05 was considered significant and 0.05 ≤ *p* < .10 trend-level for ANCOVA analyses. Effect size for each dataset was calculated as partial η^2^. Analyses were also performed without covariates (i.e., using two-tailed t-tests) with results presented in Supplementary Material. Age, education, WASI-2, segmentation, and quality control (FWHM and SNR) data were similarly compared between groups using t-tests with significance threshold *p* < .05. Sex was compared between groups using the chi-square test with threshold *p* < .05.

As an additional exploratory analysis, we examined partial correlations (controlling for %GM fraction) between metabolites and each of the core symptom domains (poverty, disorganization, and reality distortion) in patients using Spearman’s correlation coefficients with significance threshold *p* < .05. Partial Spearman’s correlations were calculated in SPSS [1]. Clinical correlations were non-parametric as core symptom domains were non-normally distributed.

## 3. Results

### 3.1. Demographic and clinical

Demographic and clinical information for participants with usable data in at least one voxel (DLPFC or VC) is presented in Table 1. No significant differences were observed between groups for age, sex, or parental education. Patients had signficantly lower years of education and WASI-2 scores.

### 3.2. DLPFC voxel excluded data

For the DPLFC voxel, of the initial sample of 46 HCs and 40 people with SZ, 3 HCs and 2 SZ were excluded for FWHM criteria, 1 HC and 1 HC for SNR criteria, and none for T%GM or CRLB criteria. From the included spectra, the NAA measurement for 1 HC was excluded for being a > 3 SD outlier.

### 3.3. DLPFC voxel analysis and clinical correlates

MRS quality and segmentation data for the remaining sample of 42 HCs and 37 people with SZ is presented in Table 2. No significant differences were observed between groups for %GM, %WM, %CSF, FWHM, or SNR measures for any metabolite. CRLB values for all metabolites except glutamate were ≤ 5. Glutamate CRLB values were all ≤ 8.

**Fig. 2.**
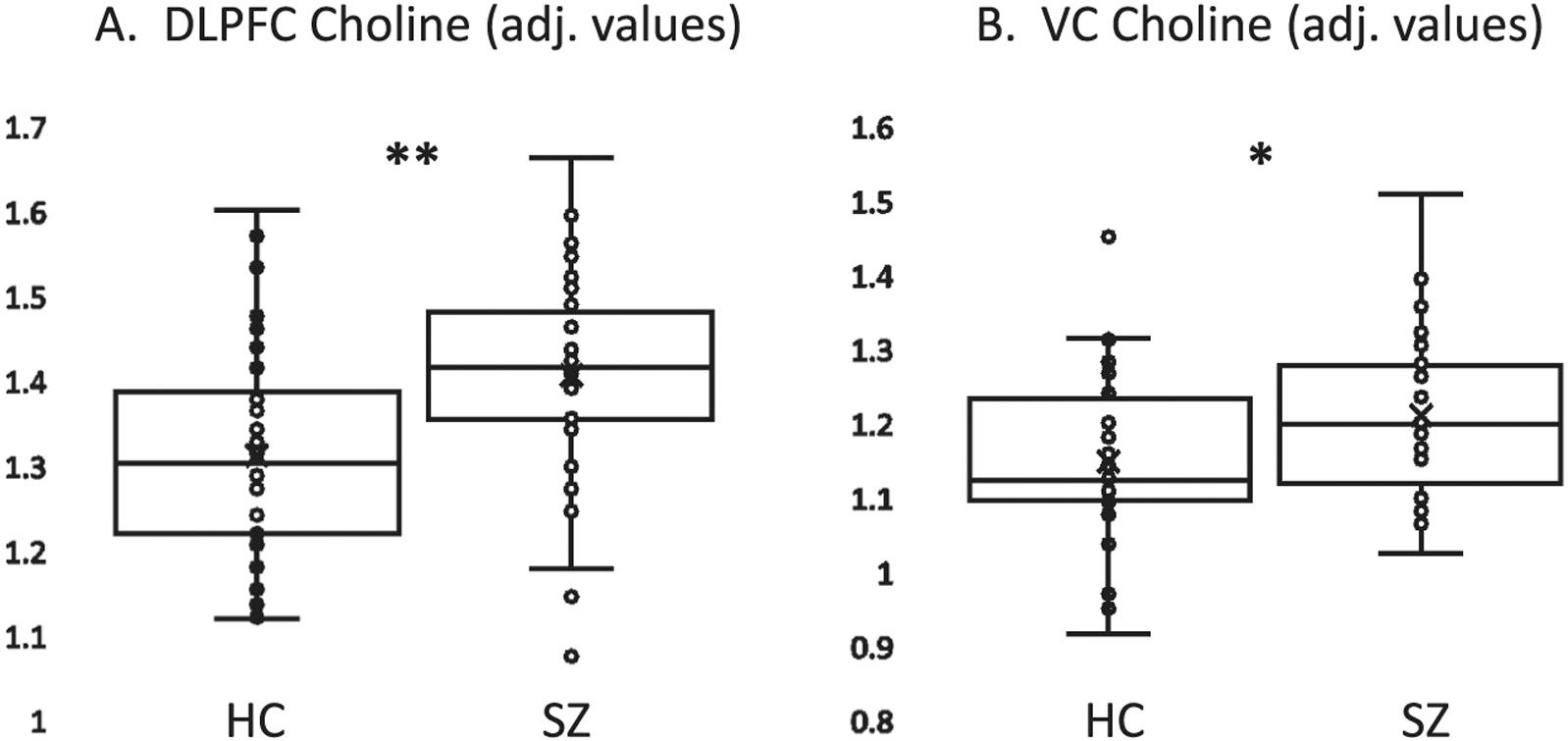
Box and whisker plots of water-normalized choline-containing compounds (institutional units, adjusted for percent gray matter in tissue fraction of voxel) showing elevation in patients with schizophrenia (SZ) compared to healthy control subjects (HC). A. Dorsolateral prefrontal cortex (DLPFC) data. B. Visual cortex (VC) data. ** p < .001; * p = .015.

Group differences in DLPFC metabolites with T%GM fraction as a covariate are also presented in Table 2. Patients with SZ had significantly higher choline content than HC for both water-normalized and creatine-normalized values (Fig. 2A). No significant effects of diagnosis were observed for the other four metabolites. Qualitatively, people with SZ and HC had similar levels of NAA and creatine, while SZ had higher myo-inositol and lower glutamate than HC, with small effect sizes. Analyses without the covariate (t-tests) yielded similar results (Supplementary Table 1).

Clinically, after controlling for T%GM fraction, a trend-level negative correlation was observed between poverty symptoms and water-normalized NAA (ρ = −0.33, *p* = .068). A trend-level negative correlation was also observed between reality distortion and water-normalized creatine (ρ = −0.32, *p* = .071). No other correlations with symptom ratings or antipsychotic dose were observed.

### 3.4. VC voxel excluded data

Of the initial sample of 44 HCs and 39 people with SZ, VC voxel data from 2 HCs and 2 SZ were excluded for FWHM criteria, none for SNR criteria, and 1 HC and 1 SZ for T%GM criteria. 1 additional HC was excluded for having a significant artifact in the spectrum. From the included spectra, the glutamate measurement for 1 HC was excluded for being a > 3 SD outlier.

### 3.5. VC voxel analysis and clinical correlates

MRS quality control information for the remaining sample of 40 HCs and 36 people with SZ is presented in Table 3. No significant differences were observed between groups for %GM, %WM, %CSF, or SNR. FWHM was significantly greater in the patient group (t = 2.60, *p* = .011). No associations, however, were observed between FWHM and levels of any metabolite. CRLB values for all metabolites except glutamate were ≤ 5. CRLB values for glutamate were all ≤ 7.

Group differences in VC metabolites with T%GM fraction as a covariate are also presented in Table 3. As in the DLPFC voxel, water-normalized choline levels were significantly higher in patients with SZ relative to HC (Fig. 2B). A trend in the same direction was observed for creatine-normalized choline. No significant effects of diagnosis were observed for any other metabolite. Qualitatively, SZ and HC had similar levels of NAA and creatine, while SZ had higher myo-inositol and lower glutamate than HC, with small effect sizes. Analyses without covariates (t-tests) yielded similar results (Supplementary Table 2).

No clinical correlations were observed with VC metabolites. No correlations with antipsychotic dose were observed.

## 4. Discussion

In this study, we examined the levels of NAA, glutamate, myo-inositol, choline, and creatine in the DLPFC and VC in HC and patients with SZ using 3 T MRS. Applying strict quality control measures to our analysis, we found a significant increase in total choline in both the DLPFC and VC in SZ. Similar elevations in choline were observed using creatine-normalized values, although only at a trend level in the VC. No significant group differences in NAA, glutamate, inositol, or creatine were observed in DLPFC or VC. Clinically, we observed trend-level negative correlations between DLPFC NAA and negative symptoms as well as between DLPFC creatine and reality distortion symptoms in the patient group.

The single metabolite that showed a significant difference between early onset SZ patients and healthy control subjects was choline, with moderate to large effect sizes for both DLPFC and VC voxels. The group difference for DLPFC choline also would be significant using a stringent Bonferroni-corrected *p* < .05/5 (for 5 metabolites)). As choline is an esssential building block of membranes, high choline levels are conventionally considered an index of membrane turnover [28] and may reflect inflammation and/or homeostatic responses to pathological processes, e.g., membrane repair [10]. Choline is also highly expressed in glial cells and may thus also represent a neuroinflammatory response associated with glial activation [33]. Our finding of significantly increased choline is thus in conceptual agreement with the viewpoint that neuroinflammatory processes may contribute to SZ pathology [18]. In contrast to our finding, 2012 and 2018 *meta*-analyses found no signfiicant differences in frontal region choline levels in SZ [16,19], although these analyses did not find a sufficient number of studies to conduct a subgroup analysis of recent onset individuals. More recent studies have reported evidence of significantly elevated choline in frontal regions in SZ [15], including one study of recent onset patients [11]. These findings will require replication in future studies before strong conclusions can be made regarding the nature of abnormalities in mobile choline-containing compounds in recent onset SZ.

We did not observe significant differences in DLPFC or VC NAA in these recent onset SZ patients. This contrasts with a recent *meta*-analysis by Whitehurst et al. [36], which reported that DLPFC NAA was significantly reduced in patients with chronic schizophrenia across 20 studies and reduced at a trend level in first episode patients across 9 studies. The pattern of NAA findings across the 9 studies of first episode patients, however, was highly heterogeneous. It is also possible that we did not observe NAA differences due to antipsychotic effects, as almost all patients in this study were medicated and previous work suggests NAA levels are higher in patients receiving neuroleptics [7]. No prior studies of VC NAA in first episode patients were found by Whitehurst et al. [36]. Exploratory clinical correlations in our patients, however, found a trend-level association between negative symptoms and NAA in the DLPFC. NAA, one of the most highly concentrated central nervous system molecules, is synthesized in neuronal mitochondria and is considered a useful indicator of neuronal integrity [26]. NAA is also involved in oligodendrocyte acetate metabolism [24], which in turn may be important for myelin synthesis [9,20]. The trend toward a negative association between NAA and negative symptoms observed in this study may thus suggest that these symptoms are related to prefrontal hypofunction or loss of white matter integrity. It is important to note that the DLPFC voxels in our study typically contained more white matter than gray matter. Thus our metabolite findings in this region may be as representative of white matter as of cortex.

In conclusion, our findings suggest that recent onset SZ is associated with significantly increased DLPFC and VC choline, possibly indicating increased membrane turnover and/or inflammatory processes in these regions.

## Supplementary Material

Supplementary Material

## Figures and Tables

**Table 1:** Demographic and clinical information for participants with usable data in at least one voxel (dorsolateral prefrontal cortex or visual cortex). Numbers in parentheses represent the standard deviation unless noted in the column heading. Abbreviations: AA = Atypical Antipsychotic, BPRS = Brief Psychiatric Rating Scale, CPZ = Chlorpromazine, GAF = Global Assessment of Function, HC = Healthy Controls, P = Typical Antipsychotic, SANS = Scale for the Assessment of Negative Symptoms, SAPS = Scale for the Assessment of Positive Symptoms, SZ = Schizophrenia, WASI-2 = Weschler Abbreviated Scale of Intelligence, 2nd Edition. *p < 0.05.

	HC (*n* = 47)	SZ (*n* = 40)	t or *χ* ^2^ (*p*)
Age	21.17 (3.35)	20.55 (3.30)	0.87 (0.39)
Sex (M/F)	32/15	27/13	0.00 (0.95)
Education Level (Years)	14.33 (2.69)	12.23 (1.90)	*4.12 (<0.001)
Parental Education Level (Years)	14.54 (3.29)	13.93 (2.84)	0.90 (0.37)
IQ (WASI-2)	115.45 (12.07)	102.74 (16.61)	*3.96 (<0.001)
Duration of Illness (Days)	—	326.49 (172.72)	
Antipsychotics (P/AA/None)	—	0/36/4	—
Antipsychotic Dose (CPZ Equivalent)	—	225.06 (187.65)	
GAF	—	45.08 (10.53)	—
Total BPRS	—	44.38 (11.66)	—
Total SANS	—	10.54 (4.07)	—
Total SAPS	—	4.00 (3.73)	—
Poverty Symptoms	—	15.00 (6.13)	—
Disorganization Symptoms	—	7.58 (3.71)	—
Reality Distortion Symptoms	—	11.61 (6.88)	—

**Table 2:** Dorsolateral prefrontal cortex voxel results in 37 patients and 42 healthy control subjects (41 for NAA). Numbers in parentheses represent the standard deviation for quality metrics and standard error for covariate adjusted metabolite values, unless otherwise noted in the column heading. Fixed effect models include precent gray matter in tissue fraction (T%GM) as a covariate, resulting in “adjusted” values. Abbreviations: COV = Coefficient of Variation, CRLB = Cramer-Rao Lower Bound, CSF = Cerebrospinal Fluid, FWHM = Full-Width Half-Max, HC = Healthy Controls, IU = Institutional Units, NAA = N-Acetyl Aspartate, SNR = Signal to Noise Ratio, SZ = Schizophrenia, WM = White Matter.

	HC Mean or Adjusted Mean	SZ Mean or Adjusted Mean	Mean COV	F_DX_ (p)	F_T%GM_ (p)	F_DX_ Partial η^2^
%GM	40.91 (5.17)	39.39 (5.76)	—	—	—	—
%WM	55.78 (5.81)	56.52 (6.43)	—	—	—	—
%CSF	3.31 (1.71)	4.09 (2.00)	—	—	—	—
Line Width FWHM	0.039 (0.004)	0.040 (0.005)	—	—	—	—
SNR	39.71 (2.84)	39.22 (2.74)	—	—	—	—
Water-Normalized						
NAA IU	8.213 (0.087)	8.226 (0.092)	0.068	0.01 (0.91)	13.34 (<0.001)	0.00
Glutamate IU	6.810 (0.083)	6.734 (0.088)	0.079	0.39 (0.53)	70.57 (<0.001)	−0.01
Myo-inositol IU	4.637 (0.056)	4.778 (0.060)	0.077	2.96 (0.09)	50.31 (<0.001)	+0.04
Choline IU	1.296 (0.019)	1.391 (0.020)	0.090	12.05 (<0.001)	0.21 (0.65)	+0.14
Creatine IU	5.560 (0.047)	5.600 (0.050)	0.055	0.34 (0.56)	62.35 (<0.001)	0.00
Creatine-Normalized						
NAA/Creatine	1.478 (0.016)	1.474 (0.017)	0.070	0.05 (0.83)	7.00 (0.010)	0.00
Glutamate/Creatine	1.225 (0.015)	1.203 (0.016)	0.081	0.93 (0.34)	8.01 (0.006)	−0.01
Myo-inositol/Creatine	0.835 (0.010)	0.854 (0.011)	0.078	1.76 (0.19)	1.84 (0.18)	+0.02
Choline/Creatine	0.234 (0.003)	0.250 (0.004)	0.090	10.71 (0.002)	28.02 (<0.001)	+0.12

**Table 3:** Visual cortex voxel results in 36 patients and 40 healthy control subjects (39 for glutamate). Numbers in parentheses represent the standard deviation for quality metrics and standard error for covariate adjusted metabolite values, unless otherwise noted in the column heading. Fixed effect models include precent gray matter in tissue fraction (T%GM) as a covariate, resulting in “adjusted” values. Abbreviations: COV = Coefficient of Variation, CRLB = Cramer-Rao Lower Bound, CSF = Cerebrospinal Fluid, FWHM = Full-Width Half-Max, HC = Healthy Controls, IU = Institutional Units, NAA = N-Acetyl Aspartate, SNR = Signal to Noise Ratio, SZ = Schizophrenia, WM = White Matter.

	HC Mean or Adjusted Mean	SZ Mean or Adjusted Mean	Mean COV	F_DX_ (p)	F_T%GM_ (p)	F_DX_ Partial η^2^
%GM	49.26 (4.60)	48.72 (4.57)	—	—	—	—
%WM	44.45 (5.11)	44.82 (5.34)	—	—	—	—
%CSF	6.28 (3.25)	6.46 (2.39)	—	—	—	—
Line Width FWHM	0.039 (0.006)	0.043 (0.006)	—	—	—	—
SNR	47.13 (4.79)	48.97 (4.70)	—	—	—	—
Water-Normalized						
NAA IU	10.712 (0.109)	10.795 (0.115)	0.064	0.27 (0.61)	1.85 (0.18)	0.00
Glutamate IU	7.993 (0.125)	7.761 (0.130)	0.099	1.66 (0.20)	5.75 (0.02)	−0.02
Myo-inositol IU	5.448 (0.068)	5.547 (0.072)	0.079	0.98 (0.32)	1.90 (0.17)	+0.01
Choline IU	1.149 (0.017)	1.210 (0.018)	0.091	6.17 (0.015)	7.90 (0.006)	+0.08
Creatine IU	7.108 (0.074)	7.136 (0.078)	0.065	0.07 (0.79)	3.21 (0.08)	0.00
Creatine-Normalized						
NAA/Creatine	1.509 (0.014)	1.516 (0.015)	0.071	0.12 (0.73)	0.23 (0.64)	0.00
Glutamate/Creatine	1.122 (0.017)	1.091 (0.019)	0.096	1.66 (0.20)	0.89 (0.35)	−0.02
Myo-inositol/Creatine	0.767 (0.008)	0.779 (0.009)	0.068	1.03 (0.31)	0.01 (0.93)	+0.01
Choline/Creatine	0.162 (0.003)	0.170 (0.003)	0.112	3.89 (0.052)	1.85 (0.18)	+0.05
