# Supplementary material for: Magnetic resonance spectroscopic evidence of increased choline in the dorsolateral prefrontal and visual cortices in recent onset schizophrenia
Source: Neurosci Lett. Author manuscript; Available in PMC 2022 Jul 28. (PMC9332487; doi:10.1016/j.neulet.2021.136410)
Supplement: Supplementary Material [file NIHMS1823466-supplement-Supplementary_Material.docx]

**
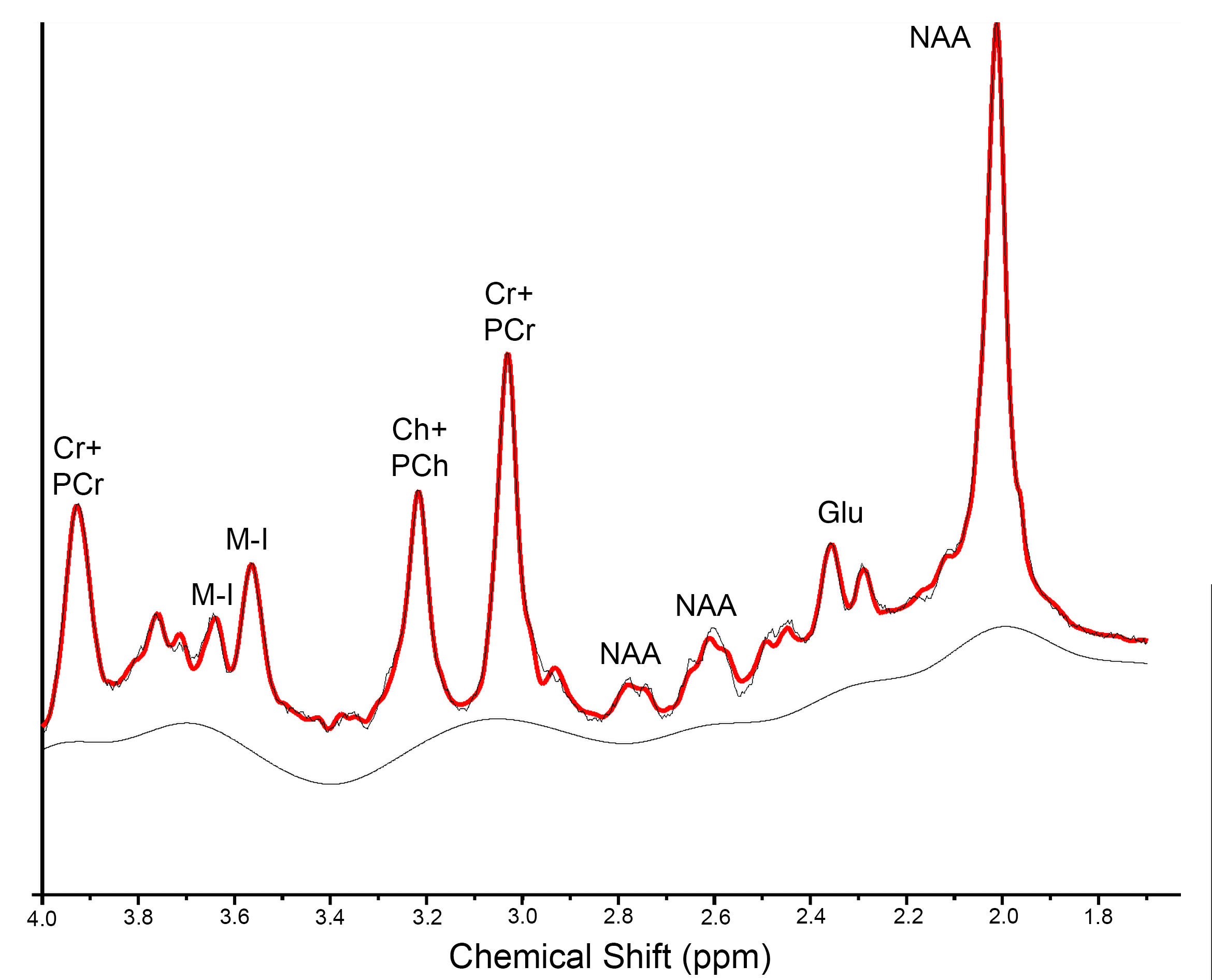
**

**Supplemental Figure:** Metabolite peaks of a representative spectrum. Abbreviations: Ch = Choline, Cr = Creatine, Glu = Glutamate, M-I = Myo-Inositol, NAA = N-Acetyl Aspartate, PCh = Phosphocholine, PCr = Phosphocreatine, PPM = Parts Per Million.

**Supplementary Table 1.** Results of two-tailed t-tests comparing dorsolateral prefrontal cortex metabolite levels in 37 patients and 42 healthy control subjects (41 for NAA). In contrast to the primary analysis, this analysis does not use gray matter fraction as a covariate. Numbers in parentheses represent the standard deviation unless otherwise specified. Abbreviations: COV = Coefficient of Variation, HC = Healthy Controls, IU = Institutional Units, NAA = N-Acetyl Aspartate, SZ = Schizophrenia.

| **DLPFC** | **HC Mean** | **SZ Mean** | **Mean COV** | **t (*p*)** | **Cohen’s *d*** |
| --- | --- | --- | --- | --- | --- |
| **Water-Normalized** |  |  |  |  |  |
| *NAA IU* | 8.237 (.492) | 8.199 (.704) | .073 | .28 (.78) | -.06 |
| *Glutamate IU* | 6.860 (.702) | 6.677 (.777) | .116 | 1.10 (.27) | -.25 |
| *Myo-inositol IU* | 4.665 (.445) | 4.745 (.482) | .098 | .76 (.45) | +.17 |
| *Choline IU* | 1.295 (.121) | 1.392 (.118) | .089 | 3.56 (.0006) | +.80 |
| *Creatine IU* | 5.587 (.364) | 5.569 (.456) | .074 | .19 (.85) | -.04 |
| **Creatine-Normalized** |  |  |  |  |  |
| *NAA/Creatine* | 1.475 (0.076) | 1.477 (.132) | .070 | .08 (.94) | +.02 |
| *Glutamate/Creatine* | 1.228 (.093) | 1.200 (.113) | .085 | 1.21 (.23) | -.27 |
| *Myo-inositol/Creatine* | .836 (.064) | .853 (.066) | .077 | 1.18 (.24) | +.27 |
| *Choline/Creatine* | .233 (.023) | .251 (.027) | .103 | 3.32 (.001) | +.75 |

**Supplementary Table 2.** Results of two-tailed t-tests comparing visual cortex metabolite levels in 36 patients and 40 healthy control subjects (39 for glutamate). In contrast to the primary analysis, this analysis does not use gray matter fraction as a covariate. Numbers in parentheses represent the standard deviation unless otherwise specified. Abbreviations: COV = Coefficient of Variation, HC = Healthy Controls, IU = Institutional Units, NAA = N-Acetyl Aspartate, SZ = Schizophrenia.

| **Visual Cortex** | **HC Mean** | **SZ Mean** | **Mean COV** | **t (*p*)** | **Cohen’s *d*** |
| --- | --- | --- | --- | --- | --- |
| **Water-Normalized** |  |  |  |  |  |
| *NAA IU* | 10.717 (.746) | 10.789 (.634) | .064 | .45 (.65) | +.10 |
| *Glutamate IU* | 7.999 (.841) | 7.755 (.760) | .102 | 1.32 (.19) | -.30 |
| *Myo-inositol IU* | 5.451 (.406) | 5.543 (.465) | .079 | .92 (.36) | +.21 |
| *Choline IU* | 1.150 (.118) | 1.209 (.107) | .096 | 2.25 (.027) | +.52 |
| *Creatine IU* | 7.112 (.429) | 7.132 (.518) | .066 | .18 (.86) | +.05 |
| **Creatine-Normalized** |  |  |  |  |  |
| *NAA/Creatine* | 1.509 (.096) | 1.517 (.085) | .060 | .38 (.71) | +.09 |
| *Glutamate/Creatine* | 1.123 (.104) | 1.090 (.107) | .095 | 1.32 (.19) | -.30 |
| *Myo-inositol/Creatine* | .767 (.042) | .779 (.059) | .065 | 1.01 (.32) | +.24 |
| *Choline/Creatine* | .162 (.019) | .170 (.017) | .109 | 1.90 (.061) | +.44 |
